# *Nos2* deficiency enhances carbon tetrachloride-induced liver injury in aged mice

**DOI:** 10.22038/ijbms.2020.39528.9380

**Published:** 2020-05

**Authors:** Deming Li, Yaping Song, Yahao Wang, Yuedong Guo, Zhaoke Zhang, Ganggang Yang, Gaiping Wang, Cunshuan Xu

**Affiliations:** 1 State Key Laboratory Cell Differentiation and Regulation, Xinxiang, Henan, China; 2 Henan International Joint Laboratory of Pulmonary Fibrosis; 3 Henan center for outstanding overseas scientists of pulmonary fibrosis, Xinxiang, Henan, China; 4 College of Life Science, Xinxiang, Henan, China; 5 Institute of Biomedical Science, Xinxiang, Henan, China; 6 Overseas Expertise Introduction Center for Discipline Innovation of Pulmonary Fibrosis (111 Project), Henan Normal University, Xinxiang, Henan, China

**Keywords:** Aged, CCl4, Knockout, Liver injury, Nos2, Oxidative stress

## Abstract

**Objective(s):**

As a multifunctional molecule, NO has different effects on liver injury. The present work aimed to investigate the effects of *Nos2* knockout (KO) on acute liver injury in aged mice treated with carbon tetrachloride (CCl_4_).

**Materials and Methods:**

The acute liver injury model was produced by CCl_4_ at 10 ml/kg body weight in 24-month-old *Nos2* KO mice and wild type (WT) mice groups. The histological changes, transaminase and glutathione (GSH) contents, and the expressions of liver function genes superoxide dismutase (SOD2) and butyrylcholinesterase (BCHE), as well as apoptosis- and inflammation-associated genes were detected at 0, 6, 16, 20, 28, and 48 hr, respectively.

**Results:**

Compared with WT aged mice, there are more fat droplets in liver tissues of *Nos2 *KO aged mice, and the serum levels of ALT and AST were elevated in the KO group; in addition, there was a decrease in the expression of SOD2 and BCHE and GSH content at multiple time-points. Furthermore, the expression of apoptosis protein CASPASE-3 was elevated from 20 to 48 hr, the same as CASPASE-9 at 28 and 48 hr and pro-apoptotic protein BAX at 6 and 28 hr, while the expression of apoptosis inhibitory protein BCL2 declined at 6 and 28 hr; at the same time the mRNA expressions of genes related to inflammation were increased at different extents in liver extracts of *Nos2* KO aged mice.

**Conclusion:**

*Nos2* KO exacerbated liver injury probably by elevated oxidative stress, apoptosis and inﬂammation response in CCl_4_-induced aged mice liver intoxication model.

## Introduction

The liver plays a critical role in the body, which has extensive physiological functions such as detoxiﬁcation, protein synthesis, regulation of blood sugar levels, and metabolism (1, 2). Liver disease is one of the most common health problems throughout the world (3), which can be induced by various factors including alcohol, viral infections, drugs abuse, and toxic chemicals (4, 5). Carbon tetrachloride (CCl_4_), a representative hepatotoxin, has been widely used to induce acute liver injury and failure in a large range of laboratory animals, where it induces triacylglycerol accumulation, oxidative stress, inﬂammation, and hepatocyte apoptosis (6, 7). In order to alleviate CCl_4_ caused liver damage, a defense mechanism involving endogenous antioxidants such as superoxide dismutase (SOD), catalase, and glutathione (GSH) has been developed by organisms (8, 9).

The elderly population is increasing day by day with life expectancy being prolonged. Aging results in the development of age-associated diseases; many organ systems undergoe reduction in the efficacy of biological function (10). Although liver’s proliferative capacity was reduced after liver damage in elderly individuals, overall liver functions still seem to remain constant (11-13). 

Nitric oxide (NO) is an omnipresent and highly diffusible messenger molecule that is involved in functional regulation in nearly all aspects of life. In mammals, NO is synthesized by three different isoforms of the nitric oxide synthase (NOS), i.e., two constitutive (nNOS or NOS1 and eNOS or NOS3) and one inducible (iNOS or NOS2) (14). *Nos2* mRNA and protein expressions were enhanced with senescence (15, 16). In the liver, NOS2-synthesized NO is protective in preventing sepsis and LPS-induced liver injury, but it may also become detrimental if produced in excess; its beneficial or detrimental effects depend on the amount, duration and the localization of NO production (17-19). 

Previous studies of liver intoxication mainly focused on young mice; this study aims to observe the effect of *Nos2* KO on CCl_4_-induced liver injury in aged mice.

## Materials and Methods

***Animals and experimental protocol***

Nos2 KO (Jackson No.: 002596) and WT control mice with C57BL/6J background were purchased from Shanghai Laboratory Animal Co. Ltd. *Nos2* KO mice were obtained as previously described (20). Mice were housed in temperature (23±3 ^°^C) and humidity (35±5%) controlled rooms with a 12-hr light/dark cycle. The experiment was divided into 3 groups: *Nos2* KO group, WT treatment group and WT blank control group. Eighteen 24-month-old *Nos2* KO mice and 18 WT mice of the same age were oral feeding with CCl_4_ at 10 ml/kg body weight [CCl_4_/olive oil (1/9, v/v)]. Three WT mice were oral feeding olive oil at 0 hr as blank control group. Serum and liver tissue were collected after CCl_4_ treatment at different time-points (0, 6, 16, 20, 28 and 48 hr, 3 mice each group). Liver injuries were detected by changes of morphology, transaminase, GSH, and gene expressions. All animal experiments complied with the Animal Protection Law of China and animal ethics.

***Histological analysis***

Liver tissues were frozen in liquid nitrogen for 30 sec and stored at -20^ °^C for 30 min, and then specimens were cut at a thickness of 7 um using a CM1850 freezing microtome (Leica Co., Germany) and stained with hematoxylin-eosin (H&E) for histological analysis under a light microscope (Nikon Eclipse TE2000-U, NIKON, Japan).

***Quantitative PCR***

Total RNA was purified from liver specimens according to the Trizol reagent specifications (Dingguo Company, China). cDNA was synthesized with reverse transcription kit using random primers (Promega). Quantitative real-time PCR was done using SYBR Green Reagent (Invitrogen, USA) in the Rotor-Gene 3000 qPCR system (Corbett Robotics). β-actin was utilized as an internal control to normalize gene expression. Genes expressions were measured by means of 2^-Δ Δ Ct ^(21). The sequence of primers used was shown in Table 1.

***Serum biochemistry***

Enzyme activities of alanine aminotransferase (ALT) and aspartate aminotransferase (AST) in plasma were assessed using the commercial kits according to the manufacturer’s instructions (Nanjing Jiancheng, China).

***Western blot analysis***

Total liver protein extracts were examined following standard Western blot procedures. The GE ImageQuant LAS400mini software was used to quantify the densities of bands. Antibodies used were: SOD2, BCHE, CASPASE-3, CASPASE-9, BCL2, BAX, and β-ACTIN. Production of the antibodies was by Boaosen China Inc. (Beijing, China).

***Measurements of glutathione content (GSH)***

Hepatic GSH content was determined in the liver homogenates after precipitation using GSH detection kit according to instruction (Beyotime, China)(22).

***Statistical analysis***

Data were presented as mean±SEM. Statistical signiﬁcance was conducted via the independent-samples T test using SPSS 19.0 software package (SPSS Inc., Chicago, USA). A *P*-value<0.05 was considered signiﬁcant.

**Figure 1:**
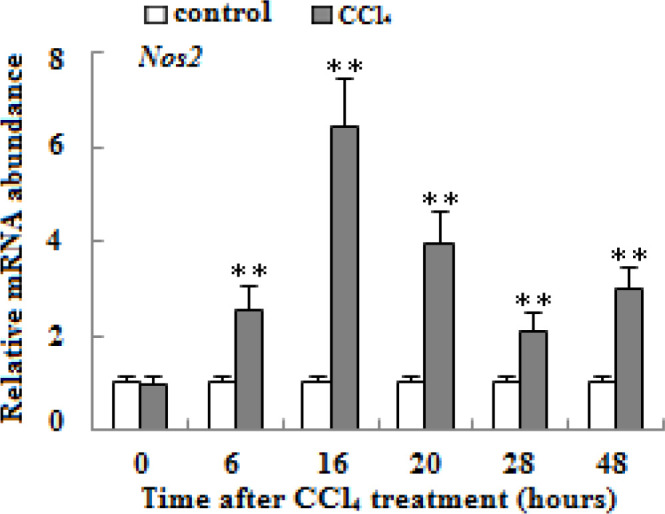
mRNA expression of *Nos2* gene in liver tissue of wild type aged mice treated with vehicle or carbon tetrachloride. mRNA level of *Nos2* was quantified by qRT-PCR methods. β-actin mRNA was used as internal control for normalization. (n=3, **means *P*<0.01)

**Table 1:** qRT-PCR primers sequence and annealing temperature

**Gene**	**Forward primer(5´- 3´)**	**Reverse primer(5´- 3´)**	**Annealing temperature**
*Nos2*	TCCTACACCACACCAAAC	CTCCAATCTCTGCCTATCC	51 °C
*TNF-α*	CGTCGTAGCAAACCACCAAGT	GGAGTAGACAAGGTACAACCCATC	58 °C
*IL-6 *	CGTGGAAATGAGAAAAGAGTTGTG	CCAGTTTGGTAGCATCCATCAT	58 °C
*IFN-γ*	TAGCCAAGACTGTGATTGCGG	AGACATCTCCTCCCATCAGCAG	58 °C
*Mcp-1*	TCAGCCAGATGCAGTTAACGC	TCTGGACCCATTCCTTCTTGG	58 °C
*Ccr2*	ATGCAAGTTCAGCTGCCTGC	ATGCCGTGGATGAACTGAGG	58 °C
*Emr1*	GGAAAGCACCATGTTAGCTGC	CCTCTGGCTGCCAAGTTAATG	58 °C
*β-actin*	CCGTAAAGACCTCTATGCCAACA	CGGACTCATCGTACTCCTGCT	58 °C

**Figure 2:**
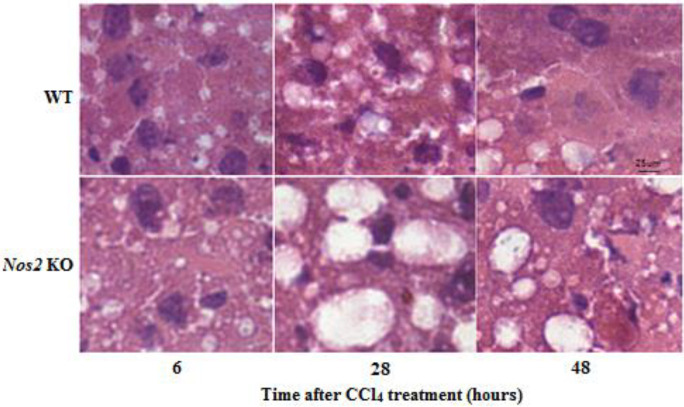
After H&E staining, liver histopathological examination of wild type and *Nos2* knockout aged mice treated with carbon tetrachloride were done under a microscope. Scale bar: 25 um; original magnification: 400×

**Figure 3:**
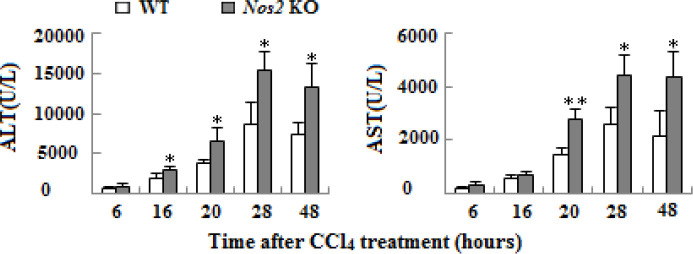
Content examination of ALT and AST in serum of wild type and Nos2 knockout aged mice treated with carbon tetrachloride at different time-points. (n=3, * means *P*<0.05, **means *P*<0.01)

**Figure 4.**
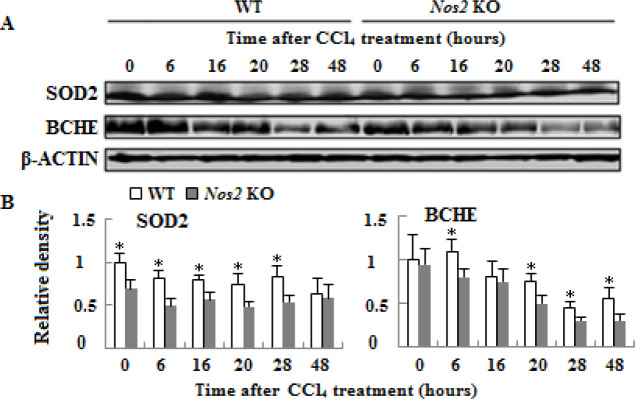
Protein expressions of liver function genes in wild type and *Nos2* knockout aged mice treated with carbon tetrachloride. (A) Western blot analysis of SOD2 and BCHE protein expression. (B) Densitometric analysis of the results shown in (A). β-ACTIN was used as control. (n=3, * means *P*<0.05)

**Figure 5:**
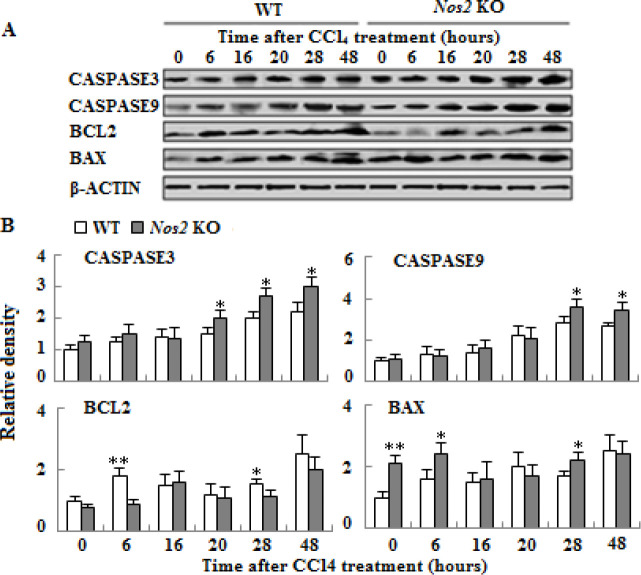
Protein expressions of apoptosis-associated genes in liver of wild type and *Nos2 *knockout aged mice treated with carbon tetrachloride. (A) Western blot analysis of CASPASE-3, CASPASE-9, BCL2, and BAX protein expression. (B) Densitometric analysis of the results shown in (A). β-ACTIN was used as control. (n=3, * means *P*<0.05, ** means *P*<0.01)

**Figure 6.**
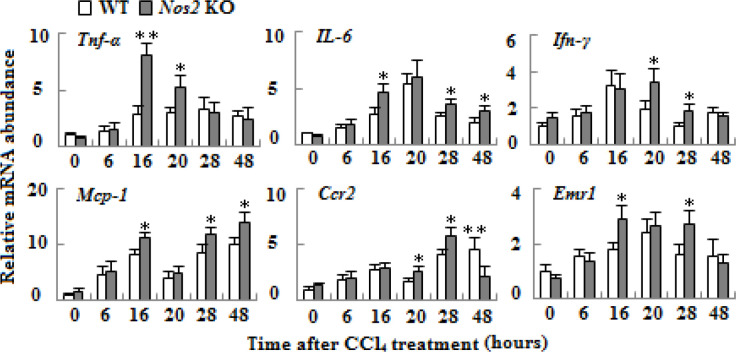
mRNA expressions of inflammation-associated genes in liver tissue of wild type and *Nos2* knockout aged mice treated with carbon tetrachloride. mRNA levels of TNF-α, IL-6, Ifn-γ, Mcp-1, Ccr2, and Emr1 were quantified by qRT-PCR methods. β-actin mRNA was used as control. (n=3, * means *P*<0.05, ** means *P*<0.01)

**Figure 7:**
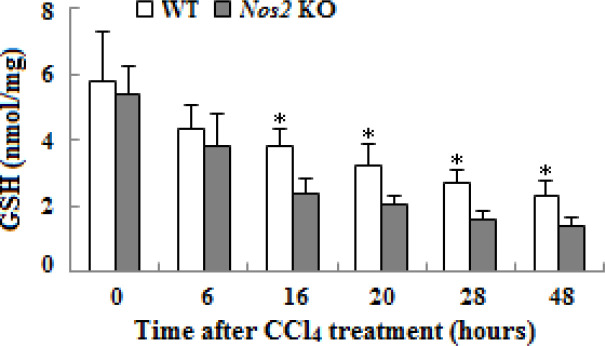
Glutathione content examination in liver tissues of wild type and* Nos2* knockout aged mice treated with carbon tetrachloride at different time-points. (n=3, * means *P*<0.05)

## Results

*Nos2* KO mice were the same as WT mice in morphology and were capable of reproducing offspring. 

***The expression of Nos2 during CCl***_4_ ***-induced acute liver injury in aged mice***

To measure the changes of *Nos2* mRNA during the course of CCl_4_-induced acute liver injury in aged mice, quantitative real-time PCR analyses of liver extracts of WT aged mice after CCl_4 _treatment were done. As shown in Figure 1 (*P*<0.01), the expression of *Nos2* mRNA in the liver was apparently increased, with peak value occurring at 16 hr.

***Liver histology***

After H&E staining, liver tissues were observed under a microscope. As shown in Figure 2, the livers in both groups revealed histological lesions characterized by cell necrosis with loss of liver cell architecture and fat droplet appearance. There were condensed nuclei and cell disintegration. *Nos2* KO mice revealed increased fat droplets and cell necrosis when compared to WT counterparts after CCl_4_ administration.

***Serum ALT and AST assay***

The serum levels of liver enzymes were analyzed to assess liver injury after CCl_4_ treatment. Compared to WT control group, there was a marked increase in serum levels of ALT and AST after 6 hr in the* Nos2 *KO group (Figure 3, *P*<0.05 or *P*<0.01).

***Nos2 KO alleviated hepatic SOD2 and BCHE expressions***

 To determine the effect of *Nos2 *KO on liver injury in CCl_4_-treated aged mice, we analyzed the protein expressions of liver function genes SOD2 and BCHE. As shown in Figure 4 (*P*<0.05), the protein expressions of SOD2 and BCHE decreased in the* Nos2 *KO group compared to the control group.

***Altered apoptosis signaling in Nos2 KO mice***

The expressions of apoptotic proteins were evaluated by Western blot analysis. Production of CASPASE-3 was higher from 20 to 48 hr, the same as CASPASE-9 at 28 and 48 hr after CCl_4_ administration in *Nos2* KO aged mice as compared to WT controls. In the Nos2 KO group a signiﬁcant increased expression of the pro-apoptotic proteins BAX at 6 hr and 28 hr and a reduced expression of the anti-apoptotic protein BCL2 at 6 hr and 28 hr were observed as compared to WT group (Figure 5, *P*<0.05 or *P*<0.01).

***Nos2 KO enhanced CCl***_4_ ***-induced activation of inﬂammation***

In order to evaluate effects of* Nos2 *KO on liver inflammation after CCl_4_ administration, the mRNA levels of inflammation-related genes including Tnf-α, IL-6, Ifn-γ, Mcp-1, Ccr2, and Emr1 were detected by quantitative real-time PCR. As illustrated in Figure 6, administration of CCl_4_ caused up-regulation of their expressions in both WT and *Nos2 *KO aged mice, but up-regulation was higher in many time-points in the* Nos2 *KO group (*P*<0.05 or *P*<0.01).

***Nos2 KO exacerbated CCl***_4_ ***-induced oxidative stress in the liver***

Antioxidant defense system in tissues involves GSH**. **We detected the GSH content in livers, which decreased markedly in both groups after CCl_4_ administration; the decline was more obvious after 16 hr in* Nos2 *KO aged mice compared to the control group (Figure 7, *P*<0.05).

## Discussion

In the present study, we investigated the effect of *Nos2 *KO on CCl_4_-induced acute hepatic injury in aged mice. CCl_4_ intoxication resulted in striking elevation of hepatic mRNA expression of the *Nos2 *gene in WT aged mice. A previous study found that the hepatic mRNA expression of *Nos2* was increased in CCl_4_-treated rats (23); its protein expression was also elevated in CCl_4_-administrated mice (24). *Nos2 *KO may contribute to enhanced hepatotoxicity in aged mice after CCl_4_ administration, as indicated by higher serum ALT and AST levels, lower protein expressions of liver function genes, and more severe histopathological changes when compared with WT aged mice. These ﬁndings suggested that *Nos2* plays a beneficial role in CCl_4_-induced liver intoxication in elderly mice. 

Oxidative stress induced by CCl_4_ plays a key role in the development of hepatotoxicity, which results in apoptosis or necrosis in liver tissues (25). To guard against the damage incurred by oxygen-free radicals, cells have developed a detoxiﬁcation and antioxidant defense system including enzymatic (e.g., SOD, CAT, and GSH-Px) and nonezymatic antioxidants such as glutathione and vitamins C and E to protect themselves from toxic injury (26). The activities of the major antioxidants SOD and GSH were measured to determine oxidative liver damage in both types of aged mice. *Nos2 *KO mice hepatic SOD and GSH activities were signiﬁcantly (*P*<0.05) decreased at multiple time-points, respectively, when compared to the control groups, which suggested that these two antioxidants were consumed in the process of antioxidant damage; the oxidative injury in the liver of *Nos2* KO aged mice was more serious. Yu *et al*. also reported that mice CCl_4_-intoxication decreased the expression of antioxidants (27). 

Previous studies have suggested that CCl_4_-administration results in severe apoptosis in rat liver (28). Caspase-3 is the chief executioner caspase of apoptosis regulated by upstream factors including Caspase-9 (6, 29).* Nos2* KO led to protein expressions of Caspase-3 and Caspase-9, which were increased at a later phase of acute liver injury compared to WT aged mice. Two major regulation proteins Bcl-2/Bax of apoptosis in the mitochondrial pathway have an important impact on cell apoptosis (29). Compared with WT aged mice, *Nos2* KO caused the expression of anti-apoptotic protein Bcl-2, which decreased at two time-points, and expression of pro-apoptotic proteins Bax was increased at three time-points. Our results suggested that *Nos2* KO increased cell apoptosis mainly after 20 hr. A previous report showed that NO can prevent Caspase-3-mediated apoptosis (30).

 Inﬂammation is induced by free radicals after CCl_4_ metabolism, with release of proinﬂammatory mediators, such as TNF-α and IL-6, which promote the progression of liver injuries (7, 31). Inflammatory signaling enhanced with aging, which potentially affected age-related changes in the liver (32, 33). CCl_4_ administration elevated hepatic expressions of inflammation-related genes in the livers of two types of aged mice. *Nos2* KO leading to mRNA expressions of these genes was higher, indicating that NOS2-synthesized NO inhibits inflammation in this model, which may alleviate liver injury. Previous investigation found *Nos2* KO elevates the expression of inflammatory mediator TNF-α and potentiates liver injury in young mice (24). Similarly, it has been reported that NO represses the expression of TNF-α in galactosamine administrated mice (34) and can relieve inflammatory injury (35).

In the liver, NO produced by NOS2 can be either protective or injurious depending on the pathological status of the liver and the amount and duration of NO production, as well as the amounts of superoxide anion at the same site (19). In the experiment we found *Nos2* KO aggravated liver injury, suggesting NOS2-synthesized NO was protective to the liver in this CCl_4_-induced elderly mice hepatotoxicity model. This is similar to previous investigation that after CCl_4_ treated cultured hepatocytes, *Nos2* is activated and NO plays a protective role by decreasing oxidative stress and inhibiting apoptosis (19). Mojena *et al.* found *Nos2* transgene alleviated mice from LPS-induced liver injury (36). Previously, two other experiments have also pointed out that NO plays a protective role in liver injury caused by CCl_4_ in rats (23, 37). NO has a beneficial effect on skin flap survival (38) and against oxidative stress in hepatocytes of catfish, Clarias magur (39). The protective effect of NO could be owing to its reaction with superoxide anion and other radicals to reduce toxic species (40, 41); NO can also decrease lipid peroxidation (37, 42); it can promote vasodilation (43) and increase hepatic arterial blood (23). In contrast, it has been reported that NO promoted liver injury in other models using different hepatotoxins (44-47). NO reacts with superoxide radical, forming a cytotoxic oxidant (peroxynitrite); peroxynitrite can not only interact with sulfhydryl residues in cell membranes resulting in lipid peroxidation, but also react with DNA, ultimately damaging the cell (45) . These investigations suggested that internal environment difference determines the beneficial or detrimental effects of NO in different models. 

## Conclusion

Our studies showed that *Nos2* KO increased hepatotoxicity in the CCl_4_-treated aged mice model, which suggested NO produced by NOS2 protects the liver against injury probably by decreasing oxidative stress, apoptosis, and inﬂammation. 
